# Estimating implicit and explicit racial and ethnic bias among community pharmacists in Canada

**DOI:** 10.1016/j.jsps.2024.102024

**Published:** 2024-03-11

**Authors:** Fahad Alzahrani, Nancy Waite, Michael Beazely, Martin Cooke

**Affiliations:** a Department of Pharmacy Practice, College of Pharmacy, Taibah University, Madinah, Saudi Arabia; b School of Pharmacy, University of Waterloo, Kitchener, Canada; c Department of Sociology and Legal Studies & School of Public Health Sciences, University of Waterloo, Waterloo, Canada

**Keywords:** Racial/ethnic bias, Implicit, Healthcare disparities, Minority health, Community pharmacist

## Abstract

**Background:**

Bias, whether implicit (unconscious) or explicit (conscious), can lead to preferential treatment of specific social groups and antipathy towards others. When healthcare professionals (HCPs), including pharmacists, act on these biases, patient care and health outcomes can be adversely affected. This study aims to estimate implicit and explicit racial/ethnic bias towards Black and Arab people among community pharmacists in Ontario, Canada.

**Methods:**

Community pharmacists participated in a secure, web-based survey using a cross-sectional design that included Harvard’s Race and Arab Implicit Association Tests (IATs) to examine bias towards Black and Arab people. Explicit (stated) preferences were measured by self-report. Data were analyzed using descriptive and inferential statistics.

**Results:**

The study surveyed 407 community pharmacists, 56.1 % of whom were women with an average age of 46.9. Implicit Association Test (IAT) results showed a statistically significant moderate preference for white people over both Black (mean IAT = 0.41) and Arab people (mean IAT = 0.35). However, most pharmacists explicitly stated that they had no racial/ethnic preference, with 75.7 % expressing a neutral preference between Black and white and 66.6 % neutral between Arab and white. However, a slight preference for white individuals was observed. Demographic factors such as age, place of birth, race/ethnicity, and experience significantly impacted IAT scores. For example, older, Canadian-born, white pharmacists with more experience displayed higher implicit bias scores. A mild correlation was found between implicit and explicit bias, indicating as implicit bias increases, explicit bias tends to become more negative.

**Conclusions:**

This study is the first to explore the issue of pharmacist bias in Canada and concentrate on anti-Arab bias. Our findings reveal that Ontario community pharmacists tend to have an unconscious inclination towards white people, which calls for further understanding of this matter. Additionally, we discovered a moderate degree of anti-Arab bias, indicating that studies on other HCPs should consider bias against this social group. Educational interventions are needed to address the implicit biases among community pharmacists in Ontario, Canada. These findings should aim to raise self-awareness of biases, educate about the potential implications of these biases on patient care, and provide strategies to reduce bias.

## 1 Introduction

Disparities in health care access and quality are evident between groups that represent different races and ethnicities. This has been well-documented across settings, diagnoses, and treatment dimensions (Braveman et al., 2011, Chauhan et al., 2020, Rockville, 2021). For example, studies have shown that minority patients are less likely than white patients to receive thrombolytic therapy, with consistent disparities in treatment observed over time (Mendelson et al., 2022, Man et al., 2024). Moreover, patients from minority backgrounds report lower satisfaction levels with their healthcare interactions, citing poor communication and a lack of patient-centered care (Hamed et al., 2022). This dissatisfaction is partly due to minorities having fewer opportunities to voice their concerns and perspectives during medical appointments, which could compromise the effectiveness of healthcare decision-making and outcomes (Shepherd et al., 2018, Hamed et al., 2022). As a result, minority patients often have less favorable views of their healthcare providers, highlighting the significant disparities that exist within healthcare experiences and results (Hamed et al., 2022).

A United States Institute of Medicine (IOM) committee report titled, *Unequal Treatment: Confronting Racial and Ethnic Disparities in Health Care,* found indirect but strong evidence of racial and ethnic discrimination at all levels of health care, from the policy level to individual and interpersonal interactions. The IOM identified healthcare provider bias as one factor that may contribute to health care disparities (Nelson, 2002). Numerous studies provide further evidence of racial and ethnic bias among healthcare providers, including implicit bias in pain treatment recommendations (Amin, 2022), lower quality communication (Johnson et al., 2004), and discrimination in recommendations for cardiac catheterization (Lawton et al., 2022). Taken together, these findings indicate that provider biases related to race and ethnicity contribute substantially to disparities across the continuum of healthcare.

For the present purposes, the term “bias” refers to a prejudiced or unsupported judgment in favor of or against one thing, person, or group compared to another in a way considered unfair or harmful. People, groups, or institutions may have biases, and their effects can be positive or negative (Wells et al., 2022). Social psychology scholars have conceptualized bias as either implicit or explicit. Explicit bias, also known as conscious bias, occurs when individuals are fully aware of their prejudices and attitudes toward particular groups. This bias manifests as intentional preferences or aversions toward specific individuals or groups, which can lead to discriminatory actions. Such biases are openly recognized and can be expressed by the individuals holding them (Killen et al., 2008). As a result, explicit bias is measurable through self-reporting methods, allowing individuals to disclose their attitudes and preferences directly (Blair and Banaji, 1996, Dovidio et al., 2002).

However, assessing explicit attitudes carries several validity concerns, notably respondent reactivity. This phenomenon occurs when individuals, aware that their attitudes are under examination, may modify them. A critical consideration in this context is the influence of social desirability bias, where respondents might provide answers they consider socially acceptable, rather than their true feelings or beliefs (Antonak and Livneh, 2000).

In contrast, “implicit” bias Implicit bias, also known as unconscious bias, is a prevalent and long-lasting type of bias that influences people's perceptions, behaviors, and decisions without their conscious knowledge. For instance, a person might unconsciously associate a particular race with criminality, leading them to be more suspicious of individuals from that race. This type of bias stems from deeply ingrained societal stereotypes and structural discrimination and affects individuals' attitudes and responses toward certain ethnic or racial groups. Cultural stereotypes, particularly those related to different racial or ethnic groups, subtly impact our interactions with members of those groups. (Fazio et al., 1995, Dovidio et al., 2002).

Implicit bias is challenging to identify and measure because it exists outside of conscious awareness and unintentionally influences actions, decisions, and understanding (Chapman et al., 2013, Hall et al., 2015, Howell et al., 2018). It can affect an individual's attitudes towards others based on their characteristics, including age, race, and ethnicity. Consequently, actions resulting from implicit bias can be difficult to identify and control. This can lead to unfair treatment, exclusion, and perpetuation of stereotypes, all of which can have serious social and psychological consequences (Hall et al., 2015).

Understanding implicit bias is crucial as it sheds light on the unconscious attitudes and stereotypes that can shape perceptions, decisions, and behaviors (Dovidio and Fiske, 2012). Marceline et al. reported that people can actively mitigate the impact of implicit bias by becoming aware of these biases and engaging in introspection (Marcelin et al., 2019). Studies have shown implicit biases can significantly affect domains such as law enforcement, criminal justice, employment decisions, education, healthcare outcomes, and interactions with marginalized groups (Zolnierek and DiMatteo, 2009, Staats, 2016, FitzGerald et al., 2019). Therefore, recognizing and addressing implicit bias fosters fairness, inclusivity, and equitable treatment of others and potentially promotes a more just and empathetic society (Greenwald and Krieger, 2006, Moskowitz and Li, 2011).

In view of the risks of response bias, researchers have developed a variety of measures for assessing implicit attitudes. A common method for measuring implicit bias is the implicit association test (IAT) (Kirwan Institute for the Study of and Ethnicity, 2018). The IAT was introduced in the scientific literature in 1998 by Greenwald et al. (Greenwald et al., 1998). This online tool available through Harvard University measures implicit preferences by avoiding conscious processing through a computerized, timed, dual categorization task (Chapman et al., 2013, Howell et al., 2018). Researchers in many disciplines, including scholars in social psychology, health, political science, and marketing have used IATs (Blair et al., 2011). In the IAT, respondents are assessed on their ability to match socially relevant concepts (e.g., age, race, gender, ethnicity) to specific attributes (e.g., cooperative, stubborn, good, bad). When subjects associate any of these concepts with an attribute, they are hypothesized to be more likely to match that attribute to a group representative. Usually, subjects are aware they are making these connections but are unable to alter them in the testing context due to the test’s design and need for fast response times (Greenwald et al., 2009).

There is extensive literature investigating bias against Black people in the U.S. context, and some studies evaluating anti-black bias among pharmacists (White-Means et al., 2009, Blair et al., 2013, Oliver et al., 2014). It is important to note that Canada’s health care system, work environments, and potentially underserved populations differ from those in the U.S. In Canada, there has been much less research attention on bias against people other than Black and Indigenous communities (Schmidt, 2020, Gran-Ruaz et al., 2022). In particular, there is a significant gap in research regarding Arab Canadians, who are among the country's quickest-growing visible minorities(Canada, 2022). Arab people in North America have been subjected to unfavorable stereotypes, especially since the 9/11 terrorist attacks (Malos, 2010). Even before 2001, Helly reported that Arab people in Canada were viewed negatively and as a potential threat (Helly, 2004).

As pharmacists' roles continue to expand in Canada, their impact on health outcomes becomes increasingly critical.(Schindel et al., 2017). Prior research has identified pharmacists and the pharmacy system as significant factors contributing to healthcare disparities. Studies have shown that disparities in access to pharmacy services lead to varied medication usage among different racial and ethnic groups, underlining inequities in healthcare provision (Pednekar and Peterson 2016). Additionally, findings reveal that elderly Black patients with chronic conditions might not be fully aware of the level of expertise pharmacists hold, partly due to less effective patient-pharmacist interactions (Youmans et al., 2007). Addressing these disparities is vital to ensure all patients have equitable access to medications and high-quality healthcare.

Given these circumstances, it is essential to examine racial or ethnic biases within the pharmacy profession. Until now, no research has delved into implicit bias among pharmacists in Canada. Furthermore, the experiences and health outcomes of Black and Arab Canadians remain largely unexamined despite their growing demographics, indicating a significant research void. There is a clear call for detailed investigations into how biases impact these groups, underscoring the need for targeted research in this area. This study aims to start addressing this gap by:

• Evaluating implicit and explicit race/ethnicity-related biases among licensed pharmacists in Ontario using established assessment tools.
• Exploring any variations in implicit and explicit bias scores across pharmacists’ demographic characteristics.
• Determining the factors that predict higher levels of implicit and explicit bias using multivariate regression analysis.
• Examining the correlation between implicit and explicit bias.

## 2 Method

### 2.1 Research design and recruitment

A cross-sectional survey was conducted among community pharmacists in Ontario, Canada, from August to December 2019. Through the Ontario College of Pharmacists, pharmacists who agreed to share their contact information for research purposes were contacted to participate in the study. A power analysis determined a sample size (n) of 372 with a 95 % confidence interval and a margin of error of 5 %.

### 2.2 Study setting

Data were collected in the central Canadian province of Ontario. It is the most populous province in Canada, with 38.3 % of the country’s population, and the second largest in terms of area (1.06 million km^2^). According to the 2021 Census of Canada, Ontario was home to 768,740 people who identified as Black, representing 49.6 % of Canada’s Black population, and 284,215 people who identified as Arab, making up 37.8 % of Canada’s Arab population (Institute, 2014, Statistics, 2017, Statistics and C , 2021).

### 2.3 Data collection

Qualtrics™ was used to develop a web-based survey. Implicit Associate Tests (IATs) were designed with Harvard Project Implicit (projectimplicit.net). After participants provided informed consent, they received a demographic questionnaire, two IATs, and explicit measure survey questions. Explicit measures were taken after the IAT to minimize the impact of answering social desirability questions on IAT responses. Study participants were informed that the study was “An Exploration of Ontario Pharmacists’ Attitudes toward Certain Social Groups.”

### 2.4 Study measures

Participant responses were collected using a structured, self-administered questionnaire, which included three sections and was developed based on the literature and direction from Harvard Project Implicit (Fitzsimmons, 2009, Blair et al., 2011, Blair et al., 2013).

#### 2.4.1 Demographic characteristics

Pharmacists were asked to provide their age, gender, place of birth, racial/ethnic background, years as a pharmacist, years as a licensed pharmacist in Canada, their highest level of education, location of practice, and the estimated number of Black and Arab people visiting their pharmacy per day.

#### 2.4.2 The implicit Association test (IAT)

Pharmacists completed two IATs: One to measure implicit bias against Black people and the other to measure implicit bias against Arab people.

In the IAT, an unconscious tendency to preferentially choose one group over another is derived from reaction times across different blocks of trials. The first test, “Race IAT,” presented images of Black and white people along with pleasant and unpleasant words as previously defined by the Harvard project. The second IAT, hereafter “Arab IAT,” presented stereotypically Arab- or European-sounding names (e.g., Mohammed, Richard) along with pleasant and unpleasant words.

In the IAT, there are seven tasks to complete (Greenwald et al., 1998). In the first block, pharmacists use response keys to categorize contrasting ideas into white images or names (right key) or Black images or Arab names (left key). “In the second block, participants are asked to go through the same procedure as before, only this time, they need to distinguish between positive and negative groups. The third block combines the tasks from the first two blocks: pharmacists are instructed to press the predetermined left key for any item categorized as white images/names or positive and the correct key for anything classified as black images/Arab names or negative.

The fourth block involves repeating the same tasks as the third block but with increased repetitions of words, images, or names. The fifth block flips the positions of the two groups from the second block. The sixth stage mirrors the third block but swaps the pairing of category-attribute combinations. The seventh and final block is similar to the fourth block, but again, with more repetitions of words, images, or names. Table 1, Table 2 provide a breakdown of each participant's seven blocks of categorization trials.

**Table 1:** Race IAT seven-task block structure.

Block	Stimuli	Items Assigned to Left Key Response	Items assigned to Right Key Response
B1	Images of Black and white people	Black image	White image
B2	Words	Positive word	Negative word
B3	Images and words	Black image or positive word	White image or negative word
B4	Images and words	Black image or positive word	White image or negative word
B5	Images of Black and White people	White image	Black image
B6	Images and words	White image or positive word	Black image or negative word
B7	Images and words	White image or positive word	Black image or negative word

**Table 2:** Arab IAT seven-task block structure.

Block	Stimuli	Items assigned to left key response	Items assigned to right key response
B1	Names of Arab or European	Arab name	European name
B2	Words	Positive word	Negative word
B3	Names and words	Arab name or positive word	European name or negative word
B4	Names and words	Arab name or positive word	European name or negative word
B5	Names of Arab or European	European name	Arab name
B6	Names and words	European name or positive word	Arab name or negative word
B7	Names and words	European name or positive word	Arab name or negative word

#### 2.4.3 Explicit bias measure

Explicit attitudes were measured through two questions about participants’ feelings towards Black, Arab, and white people. Answers to Questions 1 and 2, which asked, “Which statement best describes you?” were scaled from 1 to 7., with “1” being I strongly prefer white people over black people, “4” being neutral, and “7” being I strongly prefer Black people over white people.

### 2.5 Statistical analysis

IAT-D scores were calculated for each participant following the guidelines of Lane et al. (Lane et al., 2007) to calculate separate IAT scores for Black/white implicit bias and Arab/white implicit bias for each participant. IAT-D measures the difference in response time between contrasted conditions (e.g., white vs. Black) divided by the standard deviation of response time. A positive IAT-D score signifies a bias towards white individuals. A score ranging from 0.15 to 0.34 is representative of a mild bias, a score from 0.35 to 0.65 manifests a moderate bias, and a score of 0.66 or above demonstrates a strong bias (Lane et al., 2007).

To determine if Race or Arab implicit bias scores for community pharmacists vary based on their personal and professional characteristics, two-sample t-tests and analyses of variance (ANOVA) were conducted, followed by a Bonferroni post hoc test for multiple group comparisons. We used one-sample t-tests to investigate whether Race and Arab IAT scores differed significantly from zero. We computed Cohen’s d to determine the standardized effect size for the interpreted magnitude of implicit bias. Multivariate linear regression was used to predict Race and Arab IAT scores and identify predictors of these scores, retaining statistically significant variables in the two-sample t-tests and ANOVA. Additionally, we used Spearman’s rank-order correlation to determine whether pharmacists’ implicit bias and explicitly reported preference differed.

The significance level for all statistical tests was set at 0.05 and 2-sided. Cohen's d was used to measure the effect size of the comparison between the two means. We defined “small,” “medium,” and “large” effects as d = 0.20, 0.50, and 0.80, respectively (Cohen, 2013). All statistical analyses were performed using SPSS version 27.0.

## 3 Results

### 3.1 Community pharmacists’ demographic and practice characteristics

Four hundred and seven community pharmacists completed all three study tools (demographic questions, two IATs, and explicit direct questions). Over half of the pharmacists in the study were women (56.1 %), with an average age of 46.9 (SD = 12.2). A majority of participants (55.8 %) were born in Canada. 232 (57.0 %) pharmacists identified themselves as white/Caucasian. 72.0 % had a bachelor’s degree, and 14.6 % had a PharmD degree. More than two-thirds (68.0 %) of pharmacists practiced in urban areas. Community pharmacists have a wide range of experience, from one year to more than 40 years in practice. On a typical day in their workplace, most pharmacists (81.9 %) had direct contact with 0 to 3 patients whom they would identify as Black or Arab people. The detailed demographic and practice characteristics of the community pharmacists are shown in Table 3.

**Table 3:** Participant Characteristics and Implicit Association Test D scores for Race and Arab.

**Characteristics**	**n (%)^a^**	**IAT score^b^, mean (SD)**	
		Race	Arab
**Age (years)^c^**			
≤25	3 (0.70)	0.37 (0.49)	0.43 (0.22)
26–35	85 (20.0)	0.29 (0.45)	0.32 (0.42)
36–45	97 (23.8)	0.36 (0.38)	0.25 (0.41)
46–55	111 (27.3)	0.46 (0.34)	0.39 (0.43)
≥56	111 (27.3)	0.49 (0.39)	0.40 (0.37)
*P-value*		0.01*	0.07

**Gender**			
Man	164 (40.2)	0.41 (0.40)	0.34 (0.43)
Woman	228 (56.1)	0.40 (0.40)	0.36 (0.40)
Other	1 (0.2)	NA	NA
*P-value*		0.78	0.66

**Place of Birth**			
Canada	227 (55.8)	0.42 (0.39)	0.38 (0.38)
Other	179 (44.0)	0.39 (0.40)	0.30 (0.43)
*P-value*		0.51	0.04*

**Race/ Ethnicity Background**			
white/Caucasian	232 (57.0)	0.45 (0.36)	0.42 (0.37)
South Asian	56 (13.8)	0.35 (0.43)	0.22 (0.38)
East Asian	49 (12.0)	0.45 (0.45)	0.33 (0.42)
West Asian or Arab	46 (11.4)	0.34 (0.40)	0.14 (0.41)
Black	6 (1.5)	−0.20 (0.49)	0.63 (0.42)
Latino	1 (0.2)	NA	NA
Indigenous	1 (0.2)	NA	NA
*P- value*		0.01*	0.01*

**Highest Level of Education**			
BSc (BPharm)	291 (72.0)	041 (0.39)	0.38 (0.38)
PharmD	60 (14.6)	0.41 (0.39)	0.27 (0.42)
Masters	34 (8.3)	0.37 (0.43)	0.25 (0.47)
Doctorate level	11 (2.7)	0.27 (0.44)	0.18 (0.53)
P-value		0.81	0.17

**Practice Location^d^**			
City	276 (68.0)	0.41 (0.40)	0.31 (0.40)
Town	115 (28.0)	0.40 (0.37)	0.43 (0.40)
Village	6 (1.5)	0.30 (0.45)	0.43 (0.46)
*P-value*		0.82	0.01*

**Years as Pharmacist**			
01-Oct	116 (28.5)	0.33 (0.44)	0.33 (0.42)
Nov-20	76 (18.7)	0.34 (0.36)	0.22 (0.39)
21–30	109 (26.8)	0.46 (0.35)	0.38 (0.41)
31–40	105 (25.8)	0.49 (0.40)	0.43 (0.38)
*P-value*		0.01*	0.01*

**Years as a Licensed Pharmacist in Canada**			
01-Oct	138 (33.9)	0.31 (0.44)	0.31 (0.42)
Nov-20	75 (18.4)	0.38 (0.36)	0.23 (0.39)
21–30	98 (24.6)	0.47 (0.35)	0.41 (0.40)
31–40	88 (21.6)	0.51 (0.40)	0.44 (0.39)
*P-value*		0.01*	0.01*

**Estimated Number of Blacks or Arabs Seen per Day**			
0–3	320 (81.9)	0.40 (0.39)	0.35 (0.41)
04-Jul	66 (16.8)	0.43 (0.43)	0.34 (0.38)
07-Oct	7 (1.8)	0.17 (0.17)	0.26 (0.51)
*P-value*		0.26	0.85

*Note.*^a^Percentages do not up to 100 because of missing values and rounding. ^b^an IAT D score of 0.15 or lower means no preferences; 0.16-0.35, slight White implicit preference; 0.36–0.65, moderate White implicit preference; and higher than 0.65, strong White implicit preference. Negative scores indicate Black /Arab implicit preference with a comparable interpretation of categories. ^c^Age converted into the ordinal group. ^d^“City” was defined as 100,00 people or more, “Town” was defined as 1000 to 99,999 people, and “Village” was defined as less than 1000 people. *indicates significant at p ≤ 0.05 for *t*-test and ANOVA and is used in regression models. Abbreviation. n, number of participants; y, years; SD, standard deviation; IAT, Implicit Association Tests.

### 3.2 Implicit and explicit racial/ethnic bias

The study results, analyzed with a one-sample *t*-test, revealed statistically significant moderate preferences for white people over both Black and Arab people. The Implicit Association Test (IAT) for Race indicated a moderate preference for white people over Black people, with a mean IAT score of 0.41 (SD = 0.40, Cohen's d = 0.99). Similarly, the Arab IAT showed a moderate preference for white people over Arab individuals, with a mean IAT score of 0.35 (SD = 0.41, Cohen's d = 0.81). These results suggest the presence of racial and ethnic biases among the pharmacist participants.

**Fig. 1:**
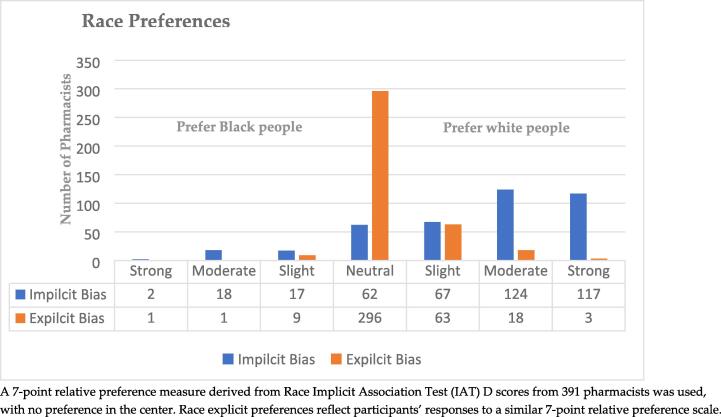
Implicit and Explicit Race Preferences among Study Pharmacists (n = 391).

**Fig. 2:**
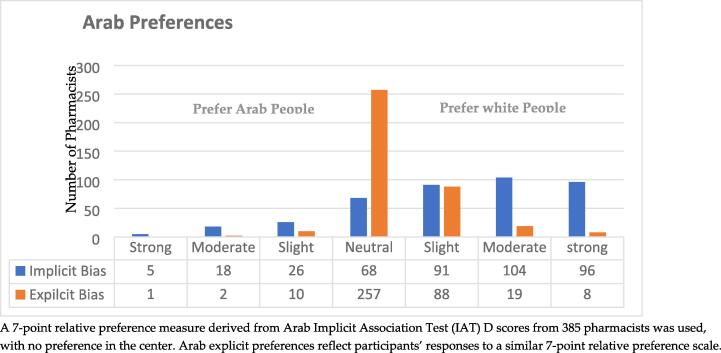
Implicit and Explicit Arab Preferences among Study Pharmacists (n = 385).

Fig. 1, Fig. 2 present the Implicit Association Test (IAT) scores range for Race and Arab tests. For the Race IAT, 78.8 % of respondents exhibited an IAT score greater than zero, indicating implicit bias favoring white people over Black people. Similarly, 75.6 % of respondents showed scores above zero for the Arab IAT, suggesting a similar implicit bias.

When considering explicit preferences, most pharmacists indicated a neutral or equal preference towards white, Black, and Arab groups. Specifically, on the Black/white preference scale, 75.7 % of participants expressed a neutral preference towards Black and white people. However, 21.4 % reported a preference for white people, with a mean score of 4.24 (SD = 0.64), suggesting a slight preference for white people over Black people.

In the context of the explicit Arab/white preference scale, the study showed a stronger explicit preference for white people over Arab individuals. 66.6 % of participants indicated a neutral preference, while 22.2 % reported a preference for white people. The mean score was 4.34 (SD = 0.75), indicating a slight preference for white people over Arabs.

### 3.3 Correlation between Race and Arab IAT scores and explicit bias

Our findings reveal a mild but significant positive correlation between implicit and explicit racial biases. Specifically, Race Implicit Association Test (IAT) scores and explicit race preference measures showed a correlation of r_s_ (391) = 0.12 with a p-value less than 0.01, implying that higher levels of implicit bias are associated with more negative explicit racial bias. The same pattern was observed for Arab IAT, where a weak positive correlation (r_s_ (385) = 0.32, p < 0.01) was found between explicit and implicit preferences, using Spearman's correlation coefficient. The findings, therefore, suggest that as implicit bias increases, explicit bias tends to become more negative, though the strength of the relationship is relatively weak.

### 3.4 Association between community pharmacists’ sociodemographic and practice variables and their Race and Arab IAT scores

The results indicate that various demographic characteristics of participants, such as age, place of birth, race/ethnicity, practice location, experience as a pharmacist, and experience as a licensed pharmacist in Canada, are significantly associated with Race and Arab Implicit Association Test (IAT) scores.

The study found a significant association between age and Race IAT scores. Precisely, younger pharmacists in the age group of 26–35 years had lower scores on the Race IAT (Mean = 0.29, SD = 0.45) compared to pharmacists aged 46–55 (Mean = 0.46, SD = 0.35), and pharmacists aged 56 and above (Mean = 0.49, SD = 0.40).

The study revealed a significant association between place of birth and Arab IAT scores. Specifically, Canadian-born pharmacists exhibited higher scores on the Arab IAT test (Mean = 0.38, SD = 0.38), indicating a higher level of implicit bias than immigrant pharmacists, who had a lower mean score (Mean = 0.30, SD = 0.43).

The study demonstrated statistically significant disparities in Race and Arab IAT scores across various self-identified races and ethnicities. In the case of the Race IAT, white pharmacists exhibited higher scores (Mean = 0.45, SD = 0.36), indicating a greater level of implicit bias, compared to Black pharmacists with a mean score of −0.20 (SD = 0.49). For the Arab IAT, the scores varied across different ethnic groups. White pharmacists had higher mean scores (Mean = 0.42, SD = 0.37) compared to South Asian pharmacists (Mean = 0.22, SD = 0.38) and West Asian/Arab pharmacists (Mean = 0.14, SD = 0.41). Interestingly, Black pharmacists had higher mean scores (Mean = 0.63, SD = 0.42) than West Asian/Arab pharmacists (Mean = 0.14, SD = 0.41). These results suggest that implicit biases, as measured by the Race and Arab IAT, can vary statistically significantly across different racial and ethnic groups within the same profession. (Table 3).

The location of practice also statistically significantly influenced Arab IAT scores. Pharmacists practicing in towns exhibited higher scores on the Arab IAT (Mean = 0.43, SD = 0.40), indicating a higher level of implicit bias, compared to those practicing in cities, who had a lower mean score (Mean = 0.31, SD = 0.40) (Table 3).

The study found that years of experience as a pharmacist had a statistically significant relationship with both Race and Arab IAT scores. For the Race IAT, more experienced pharmacists exhibited higher mean scores (Mean = 0.49, SD = 0.39), indicating a higher level of implicit racial bias than less experienced pharmacists (Mean = 0.32, SD = 0.44). The Arab IAT scores also varied with years of experience. Pharmacists with 11–20 years of experience had statistically significantly lower scores (Mean = 0.22, SD = 0.40) than those with 21–30 years of experience (Mean = 0.39, SD = 0.40).

Lastly, the study found a statistically significant relationship between the years as a licensed pharmacist and Race and Arab IAT scores. In terms of the Race IAT, pharmacists with 1–10 years of experience had lower scores (Mean = 0.30, SD = 0.42), indicating a lower level of implicit racial bias as compared to those with 21–30 years (Mean = 0.48, SD = 0.34) and 31–40 years of experience (Mean = 0.51, SD = 0.40). Similarly, for Arab IAT scores, pharmacists with 1–10 years of experience had statistically significantly lower scores (Mean = 0.30, SD = 0.41) than those with 31–40 years of experience (Mean = 0.45, SD = 0.38) (Table 3.).

### 3.5 Multivariate regression between community pharmacists characteristics and Race and Arab IAT scores

A multiple regression analysis was used to predict race IAT scores from age, race/ethnicity, and years of being licensed as a pharmacist in Canada. The overall regression model was statistically significant, F(7, 391) = 5.01, p < 0.01, and accounted for approximately 8 % of the variance in Race IAT scores (adj. R^2^ = 0.08).

Of the variables examined, age and race/ethnicity (Black pharmacists specifically) were significant predictors of Race IAT scores. Age had a positive relationship, whereby each additional year of age was associated with a 0.01 increase in predicted Race IAT score, controlling for the other variables. Compared to white pharmacists, Black pharmacists had significantly lower predicted Race IAT scores, with a coefficient of −0.63, p < 0.01. Years licensed as a pharmacist in Canada group was not a significant predictor in the model. The other race/ethnicity variables (East Asian, South Asian, West Asian/Arab) were also non-significant (Table 4 and Table 5).

**Table 4:** Multivariable Regression Model between Community Pharmacists and Race IAT Scores (N = 399 Pharmacists in models).

Variables	Unstandardized Coefficient		Standardized Coefficient	Sig.	95 % CI	
	B	Std. Error	Beta		Lower	Upper
Constant	0.16	0.11		0.27		
Age	0.01	0.00	0.22	0.03*	0.00	0.01
Years as a licensed pharmacist in Canada	−0.01	0.00	−0.03	0.74	−0.08	0.06
Race/Ethnicity^a^						
Black	−0.63	0.16	−0.19	0.00*	−0.95	−0.31
East Asian	−0.06	0.06	0.04	0.49	−0.08	0.17
South Asian	−0.05	0.06	−0.05	0.36	−0.18	0.06
West Asian/Arab	−0.08	0.07	−0.06	0.25	−0.21	0.05

*Note.* Whites are the reference group for race/ethnicity. All relationships were non-significant except for age. The model was adjusted for age, years as a licensed pharmacist in Canada, and race/ethnicity. * indicates significant at p ≤ 0.05.

**Table 5:** Multivariable Regression Model between Community Pharmacists and Arab IAT Scores (N = 398 Pharmacists in models).

Variables	Unstandardized Coefficient		Standardized Coefficient	Sig.	95 % CI	
	B	Std. Error	Beta		lower	upper
Intercept	0.34	0.12		0.01	0.11	0.57
Age	−0.02	0.04	−0.07	0.53	−0.01	0.11
Years as a licensed pharmacist in Canada	0.04	0.04	0.12	0.27	−0.03	0.01
Race/Ethnicity^a^						
Black	0.23	0.17	0.07	0.17	−0.10	0.56
East Asian	−0.12	0.07	−0.06	0.25	−0.22	0.06
South Asian	−0.19	0.07	−0.16	0.01*	−0.33	−0.05
West Asian/Arab	−0.28	0.08	−0.22	0.01*	−0.44	−0.12
Place of Birth	0.06	0.05	0.08	0.23	−0.04	0.17
Practice Location^b^						
Town	0.06	0.04	0.07	0.18	−0.03	0.15
Village	0.10	0.16	0.03	0.53	−0.22	0.43

*Note.*^a^white category is the reference group for race/ethnicity. ^b^City category is the reference group for practice location. * indicates significant at p ≤ 0.05.

A multiple regression model was conducted to predict Arab IAT scores from demographics (age, race/ethnicity, place of birth), years licensed as a pharmacist, and practice location. The overall model was statistically significant, F (11, 386) = 3.40, p < 0.01, and accounted for approximately 9 % of the variance in Arab IAT scores (adj. R^2^ = 0.09).

Of the variables examined, the race/ethnicity categories of West Asian/Arab and South Asian pharmacists were significant predictors. West Asian/Arab pharmacists had significantly lower predicted Arab IAT scores compared to white pharmacists, with a coefficient of −0.28 and p < 0.01. Similarly, South Asian pharmacists had significantly lower predicted Arab IAT scores than white pharmacists, with a coefficient of −0.19 and p < 0.01. The other demographic variables, such as years of license and practice location, were not significant predictors in the model.

## 4 Discussion

The study provides revealing insights into the implicit and explicit biases among community pharmacists towards racial and ethnic minorities. Despite the majority of participants explicitly indicating no preference towards racial groups, the Implicit Association Test (IAT) scores demonstrated a significant moderate preference for white people over Black and Arab individuals. These results underscore the prevalence of implicit bias, often diverging from individuals' self-reported explicit attitudes. Similarly, international studies have found that HCPs, including pharmacists, favor whites over Blacks (Hall et al., 2015, FitzGerald and Hurst, 2017, Maina et al., 2018, Santee et al., 2022).

It is essential, however, to note that while implicit biases can affect our initial reactions or automatic responses, they do not necessarily determine our actual behavior (Kawakami et al., 2005). Numerous studies have explored the relationship between implicit bias and behavior, and the findings are mixed. Some studies have shown a weak-to-moderate correlation between implicit bias and discriminatory behaviors, while others have found little to no association (Oliver et al., 2014, Haider et al., 2015a, Haider et al., 2015b). Therefore, it is crucial to acknowledge that implicit biases are only one among several factors that can affect behavior, and their influence may vary based on contextual and individual factors (Ajzen, 1991). Nonetheless, it is crucial to raise awareness about implicit biases and work towards reducing their impact through education, consciousness, and diversity and promoting inclusive behaviors and policies (Ajzen, 1991, Onyeador et al., 2021).

In terms of explicit (conscious) preferences, 15.2 % of community pharmacists did not express a clear preference towards white or Black individuals, and 16.5 % did not express a clear preference towards white or Arab individuals. This implies that, on a conscious level, these pharmacists do not favor one racial or ethnic group over another. This is an important finding as it provides insight into the percentage of the pharmacy population that does not harbor implicit biases against these groups, which can be a critical factor in tackling healthcare disparities. However, explicit bias is only part of the picture. Implicit biases and social desirability bias can still impact behavior and decision-making (Devine et al., 2012).

The demographic and practice characteristics of the participants further elucidated the factors that may influence these biases. For example, age was found to be associated with Race IAT scores. Older pharmacists demonstrated higher IAT scores, suggesting increased implicit bias with age. This could be due to generational differences in exposure to diversity and attitudes towards race (Gonsalkorale et al., 2009). It may also explain the lower levels of bias among younger people. Because Black people, in particular, were portrayed more negatively in the past, older people may have stronger racial/ethnic biases than younger people (Danigelis and Cutler, 1991, Wilson and Yoshikawa, 2007). However, Gonsalkorale et al. stated that older individuals respond with prejudice not because they have more biased associations than younger individuals but because they struggle to suppress their associations more (Gonsalkorale et al., 2009).

Interestingly, years of experience as a pharmacist and years as a licensed pharmacist seemed to affect the IAT scores. It was found that pharmacists who had more experience and had been practicing for a more extended period of time had higher scores on the Race and Arab IAT, indicating that implicit bias may increase over time in the profession. Our findings differ from those of FitzGerald et al., who found that physicians' implicit bias toward patients with mental illness was significantly positively influenced by their level of experience (FitzGerald et al., 2022). This result could be due to differences in education, training, or societal changes over time (Girod et al., 2016). This finding highlights the need for further research as it may have implications for bias training and interventions in the pharmacy field.

There was also a difference in the strength of Arab IAT scores based on participants’ place of birth. Foreign-born community pharmacists had less implicit bias than those who were locally born. This is unsurprising given the high proportion of racialized people among immigrants to Canada. One study suggests that immigrants may use their personal experiences or those of their families as reference points when responding to questions about social group preferences, which could potentially reduce implicit bias against racialized groups (Kolbe and Crepaz, 2016).

The Race IAT results showed that Black pharmacists implicitly favored Black people over white people. This could be a manifestation of ingroup bias, where individuals show a preference for their own social or racial groups. However, when evaluated with the Arab IAT, Black pharmacists exhibited a strong preference for white people over Arab people. This demonstrates that implicit biases can vary greatly depending on the specific groups being compared and that biases are not uniform across different racial and ethnic groups.

The finding that West Asian and Arab pharmacists displayed a preference for white people over Arab people is indeed surprising and contradicts the common phenomenon of ingroup favoritism. However, this kind of result can occur due to various factors and is supported by previous research. One explanation might be related to the status of the ingroup relative to other groups in society. A study found that members of low-status groups tended to show less ingroup preference than members of high-status groups and might even display out-group preference. This occurs when ingroup members perceive their group as being inferior to other groups, which can lead to a preference for the higher-status out-group (Clark and Clark, 1996).

Another explanation could be the “black sheep effect,” a phenomenon in which individuals negatively judge ingroup members who are perceived as acting in a way that threatens the positive image and identity of the group (Marques et al., 1988). In such cases, individuals may distance themselves from their ingroup and prefer an out-group. This refers to a situation in which an individual within one’s group is seen acting in a way that threatens the image and identity of the group as a whole (Pinto et al., 2010). These findings highlight the complexity of implicit biases and the multiple factors that can influence them. It underscores the importance of a nuanced understanding of these biases, especially in healthcare, where they can impact the quality of care delivered to diverse patient populations.

Consistent with the literature (Jin et al., 2016, Vuletich et al., 2023), the study found that the geographical location of practice significantly influenced Arab IAT scores, with pharmacists practicing in towns displaying higher scores than those in cities. Urban areas, such as cities, often have a greater level of racial and ethnic diversity compared to smaller towns. This diversity can lead to more frequent interactions between individuals of different racial and ethnic backgrounds, which can help to reduce biases (Bai et al., 2020). This finding underscores the potential role of environmental factors, such as geographical location and level of diversity, in influencing implicit biases.

## 5 Interventions for addressing implicit bias in community pharmacists

Like other healthcare professionals, community pharmacists are not immune to implicit bias. These subconscious attitudes can have far-reaching effects on patient interactions and treatment outcomes, particularly for marginalized groups. The good news is that research indicates that interventions such as perspective-taking, counter-stereotyping, and individuation can help reduce implicit bias. Forscher et al.'s meta-analysis found that interventions providing individuals with strategies to recognize and challenge their biases yielded promising results (Forscher et al., 2019). Similarly, Devine and colleagues developed a multi-faceted prejudice habit-breaking intervention that proved effective in long-term reductions in implicit racial bias. The intervention combined awareness of implicit bias, concern about its consequences, and strategies for reducing bias (Devine et al., 2012).

One study exposed participants to counter-stereotypes, showing positive examples of minority identities (e.g., Denzel Washington) and negative examples of white identities (e.g., Timothy McVeigh). This method resulted in a 50 % reduction in unintentional racial bias, which persisted 24 h after the task was finished (Dasgupta and Greenwald, 2001). Rudman et al. conducted a study in which white students enrolled in a prejudice and conflict seminar showed significantly reduced implicit and explicit anti-Black biases compared to control students. The authors proposed that affective processes may effectively instigate these changes (Rudman et al., 2001). Phelan et al. reported that training in treating minorities, improving diversity climates, less harmful role modeling, and more positive interactions with social minorities during medical school could improve medical student bias towards minorities (Phelan et al., 2017). Finally, cultural competency training is a crucial intervention in healthcare that can enhance patient outcomes and minimize health disparities, as evidenced by a systematic review that found a positive correlation between cultural competency training and improved patient outcomes (Lie et al., 2011). Therefore, US and Canadian pharmacy schools offer varying degrees of content on health disparities, cultural competence, and health literacy (Chen et al., 2021). Although the primary driver of this initiative is to increase indigenous competencies, such courses may have positive effects on reducing biases across the board.

The findings from this study exploring implicit and explicit racial/ethnic biases among pharmacists in Ontario, Canada can have important implications. Documenting the degree of bias against Black and Arab individuals provides valuable insights that may inform policy, education, and practice changes aimed at promoting health equity. Specifically, evidence of bias could highlight the need for improved training, awareness-raising, and debiasing interventions within pharmacy education and continuing professional development programs. Additionally, a better understanding of how demographics may correlate with higher bias can guide tailored approaches to addressing disparities. More broadly, this research underscores the importance of ongoing critical examination of attitudes, assumptions, and biases within healthcare to foster truly patient-centered, culturally-sensitive, and equitable care.

## 6 Strengths and weaknesses

This research is the first to examine pharmacists’ bias in Canada and focus on anti-Arab bias. Limitations include that attitudes may have changed since our data were collected and that social desirability may have resulted in some respondents altering their responses to the explicit bias questions in light of the study's objective. This study does not address the correlation between community pharmacists' implicit racial/ethnic biases and pharmacists' behaviors or actual health disparities. Lastly, the instruments have been criticized for their test–retest reliability despite the IAT's good internal consistency. There is some question as to whether the IAT measures stable implicit attitudes or if other nonattitudinal factors influence performance (Rezaei, 2011, Welsch et al., 2021).

## 7 Conclusion

We find evidence of unconscious bias against Black and Arab people among community pharmacists in Ontario. This finding is significant because biases in healthcare settings can contribute to healthcare disparities, including differential treatment and access to care for marginalized groups.

## 8 Author contributions.

All authors contributed to the design and conduct of the study, interpretation of the findings, and edited drafts of the article. F. Alzahrani wrote the initial draft of the article and conducted the data analysis.

## 9 Funding.

The authors thank the 10.13039/100018948Saudi Arabian Cultural Bureau in Canada for funding a portion of this research work through project number SACB 36582.

## 10 Institutional review board statement

The research protocol, including informed consent procedures, received full ethical review and approval from the University of Waterloo Research Ethics Committee (ORE # 22430).

## 11 Informed consent statement

Informed consent was obtained from all Pharmacists involved in the study.

## CRediT authorship contribution statement

**Fahad Alzahrani:** Conceptualization, Funding acquisition, Data curation, Writing – original draft, Writing – review & editing, Visualization, Investigation, Validation, Formal analysis, Methodology, Supervision, Project administration, Software. **Nancy Waite:** Conceptualization, Funding acquisition, Data curation, Writing – original draft, Writing – review & editing, Validation, Methodology, Supervision, Resources. **Michael Beazely:** Data curation, Writing – original draft, Writing – review & editing. **Martin Cooke:** Data curation, Writing – original draft, Writing – review & editing, Formal analysis.

## Declaration of competing interest

The authors declare that they have no known competing financial interests or personal relationships that could have appeared to influence the work reported in this paper.
